# The effect of *UGT1A9*, *CYP2B6* and *CYP2C9* genes polymorphism on individual differences in propofol pharmacokinetics among Polish patients undergoing general anaesthesia

**DOI:** 10.1007/s13353-016-0373-2

**Published:** 2016-11-08

**Authors:** Adam Mikstacki, Oliwia Zakerska-Banaszak, Marzena Skrzypczak-Zielinska, Barbara Tamowicz, Michał Prendecki, Jolanta Dorszewska, Marta Molinska-Glura, Malgorzata Waszak, Ryszard Slomski

**Affiliations:** 1 Department of Anaesthesiology and Intensive Therapy, Regional Hospital, Juraszow 7/19, 60-479 Poznan, Poland; 2 Institute of Human Genetics, Polish Academy of Sciences, Strzeszynska 32, 60-479 Poznan, Poland; 3 Laboratory of Neurobiology, Poznan University of Medical Sciences, Przybyszewskiego 49, 60-355 Poznan, Poland; 4 Department of Computer Science and Statistics, Poznan University of Medical Sciences, Dabrowskiego 79, 60-529 Poznan, Poland; 5 Department of Functional Anatomy, University School of Physical Education in Poznan, Krolowej Jadwigi 27/39, 61-871 Poznan, Poland; 6 Department of Biochemistry and Biotechnology, University of Life Sciences, Dojazd 11, 60-632 Poznan, Poland

**Keywords:** Propofol, Individual response, Pharmacokinetics, General anaesthesia, Genotyping

## Abstract

Propofol (2,6-diisopropylphenol) is one of the safest and most commonly used anaesthetic agents for intravenous general anaesthesia. However, in clinical practice, a large inter-individual variability in response to propofol is observed. To limit the risk of adverse effects, pharmacogenetic investigations are recommended. The aim of our study was to verify the impact of genetic changes c.516G>T in the *CYP2B6*, c.98T>C in the *UGT1A9* and c.1075A>C in the *CYP2C9* genes on the individual propofol pharmacokinetic profile in the Polish patients undergoing general anaesthesia. Eighty-five patients from the Department of Anaesthesiology and Intensive Therapy, Regional Hospital in Poznan, Poland, anaesthetised with propofol for surgery, were enrolled in the study. We have genotyped *CYP2B6*, *UGT1A9* and *CYP2C9* polymorphisms with the use of pyrosequencing. HPLC measurements of propofol plasma concentration were applied for a pharmacokinetic analysis of the anaesthetic. We identified poor (20), intermediate (42) and rapid (23) metabolisers of propofol, which constituted 24%, 49% and 27% of the group, respectively. Homozygotes c.516 T/T in the *CYP2B6* gene were statistically more often found in the rapid metabolisers group (*p* < 0.05). However, polymorphisms c.98T>C in the *UGT1A9* and c.1075A>C in the *CYP2C9* genes did not affect the pharmacokinetic profile of propofol. The mean propofol retention time (MRT) correlated with the patient’s body mass index (BMI) (*p* < 0.05). From all the analysed changes, only polymorphism c.516G>T in the *CYP2B6* gene and BMI affect the metabolism rate of propofol and may play an important role in the optimisation of propofol anaesthesia.

## Introduction

Propofol is one of the safest and most commonly used anaesthetic agents for intravenous general anaesthesia. However, in clinical practice, a large inter-individual variability, including adverse reactions, is observed in response to this anaesthetic (Pasin et al. 2015). Changes between individuals in the pharmacokinetics of propofol result in differences in the required dose of anaesthetic needed for efficient general anaesthesia (Karwacki et al. 2014). This variability is mostly assigned to the genetic polymorphism of genes coding for enzymes participating in the biotransformation pathway of propofol (Kübler 2005; Mikstacki et al. 2013). The need for gene profiling in anaesthesia has been suggested many times recently (Landau et al. 2012).

Propofol is metabolised mainly in the liver by cytochrome P450 2B6 (CYP2B6) and cytochrome P450 2C9 (CYP2C9) or by UDP-glucuronosyltransferase 1A9 (UGT1A9) (Restrepo et al. 2009).

UGT1A9, playing a key role in the biotransformation of propofol, is responsible for conjugation with glucuronic acid of around 70% of the metabolised anaesthetic. Because the enzyme present in the liver, kidney, colon, ovary and testis is involved in the elimination process of important drugs, such as irinotecan and flavopiridol, the polymorphism of the *UGT1A9* gene is a subject of pharmacogenetic studies. Among the most essential variants of the *UGT1A9* gene, leading to decreased enzyme activity, are three known amino acid changes: p.M33T, p.D256N and p.Y242X. Sequence variation in codon 33 (c.98T>C, rs72551330, UGT1A9*3) was identified previously in the Polish population with an allele C frequency of 0.016 (Zakerska et al. 2013). This substitution is defined as affecting the pharmacokinetic profile and catalytic efficiency of binding propofol to UGT1A9 (Korprasertthaworn et al. 2012). An association of this variant with a reduced glucuronidation level and liver failure in patients treated with entacapone and irinotecan was observed (Villeneuve et al. 2003; Martignoni et al. 2005).

CYP2B6 and CYP2C9, catalysing hydroxylation of propofol in humans, participate in the biotransformation of a wide range of drugs. A variable expression level of these enzymes due to a highly polymorphic nature of genes *CYP2B6* and *CYP2C9* makes them relevant pharmacogenes. In the context of propofol response, the most common single nucleotide polymorphism (SNP) c.516G>T (p.Q172H, rs3745274) in exon 4 of the *CYP2B6* gene was analysed in several investigations. The effect of this SNP was proved to be substrate-specific, and usually led to a disturbed gene expression.

For the *CYP2C9* gene, over 65 haplotypes have been described, including insertions, deletions and substitutions (http://www.cypalleles.ki.se/cyp2c9.htm). In global studies, two non-synonymous changes, p.R144C (c.430C>T, rs1799853, CYP2C9*2) and p.I359L (c.1075A>C, rs1057910, CYP2C9*3), determining a poor metabolising phenotype, are intensively analysed. A substrate-dependent decrease in the activity of this enzyme may occur. Rare alleles, CYP2C9*6 (c.818delA, rs933213) resulting in a lack of enzyme activity and allele CYP2C9*4 (p.I359T), have been identified in patients suffering from side effects after phenytoin application (Restrepo et al. 2009).

Awareness of the consequences of important changes in the *UGT1A9*, *CYP2B6* and *CYP2C9* genes in response to propofol would make it possible to increase the safety of patients undergoing general intravenous anaesthesia. The aim of this study was to verify the impact of genetic changes c.516G>T in the *CYP2B6*, c.98T>C in the *UGT1A9* and c.1075A>C in the *CYP2C9* genes on the individual propofol pharmacokinetic profile in the Polish patients under general anaesthesia.

## Materials and methods

### Patients

Eighty-five Polish patients (32 women and 53 men) undergoing propofol general anaesthesia (10 mg/mL propofol injectable emulsion; Diprivan, AstraZeneca, Macclesfield, UK) for laryngological surgery in the Department of Anaesthesiology and Intensive Therapy, Regional Hospital in Poznan, Poland, were enrolled in this study. All participants gave their informed consent. No history of addiction to alcohol or nicotine of patients was reported. Patients involved in the study represented classes I and II of the American Society of Anesthesiologists (ASA) scale. The study was approved by the Ethical Committee of the Poznan University of Medical Sciences, Poznan, Poland (resolution no. 653/09).

Anaesthesia was induced with propofol (2 mg/kg), followed by a continuous infusion at the rate of 8 mg/kg/h plus boluses (20–30 mg). Additionally, fentanyl was used to maintain anaesthesia. The infusion time, total dose of propofol, sex, age and body mass index (BMI) were monitored. The characteristics of the patient group are shown in Table 1.

**Table 1:** Characteristics of the patient group, with clinical parameters

Parameter		
Sex	Women	32
	Men	53
Age	Mean	44.3
	Range	31–56
BMI	Mean	27
	Range	20.1–44.8
Total dose of propofol (mg)	Mean	691.4
	Range	130–2200
Infusion time (min)	Mean	47
	Range	10–145

All subjects were screened for plasma propofol concentration in five time points as follows: at the end of anaesthesia and 5, 10, 20 and 30 min later. The whole study group was also genotyped for *UGT1A9*, *CYP2B6* and *CYP2C9*.

### Molecular analysis

Genomic DNA was isolated from the peripheral blood of all participants using the method with guanidine isothiocyanate (GTC). Three polymorphic changes, p.Q172H (c.516G>T) in the *CYP2B6*, p.M33T (c.98T>C) in the *UGT1A9* and p.I359L (c.1075A>C) in the *CYP2C9* genes, were analysed using pyrosequencing. The amplification and genotyping conditions of a *UGT1A9* gene fragment have been published previously (Zakerska et al. 2013). The PCR procedure of fragments containing codons 172 of the *CYP2B6* and 359 of the *CYP2C9* genes was carried out in a total volume of 30 μL using 0.75 U of FIREPol® DNA Polymerase, 2.5 μL 10× buffer, 2.0 μL dNTP mix (2.5 mM each dNTP), 1.5 mM MgCl_2_ solution, 80 ng DNA and 0.2 μM of each primer (Table 2). Amplification involved 50 cycles at 95 °C for 30 s, 60 °C for 30 s and 72 °C for 60 s. All reagents were obtained from Solis BioDyne (Tartu, Estonia). The PCR products were analysed in 1.5 % agarose gels electrophoresis. Pyrosequencing was performed by the PSQ™ 96MA system (Qiagen) using PyroMark™ Gold Q96 Reagents (Qiagen GmbH, Hilden, Germany), as described by the manufacturer.

**Table 2:** Primers used for the amplification and pyrosequencing of the *CYP2B6* and *CYP2C9* genes

	Direction	Primer name	Sequence	Product length
Amplification	Forward (*)	CYP2B6_Q172Hf	5′-CCTGCTGCTTCTTCCTAGGG-3′	83 bp
	Reverse	CYP2B6_Q172Hr	5′-GACGATGGAGCAGATGATGTTG-3′	
	Forward (*)	CYP2C9_I359Lf	5′-ATGCAAGACAGGAGCCACATG-3′	181 bp
	Reverse	CYP2C9_I359Lr	5′-GGGACTTCGAAAACATGGAGTTG-3′	
Pyrosequencing	Reverse	CYP2B6_Q172Hseq	5′-TGATGTTGGCGGTAAT-3′	
	Reverse	CYP2C9_I359Lseq	5′-TGGGGAGAAGGTCAA-3′	

(*) = primers labelled with biotin

### Pharmacokinetic analysis

Propofol concentration in plasma samples was measured using the HPLC/UV system (P580A; Dionex, Germany) coupled to a fluorescence detector (RF2000; Dionex, Germany) detector. As an analytical standard, propofol obtained from Toronto Research Chemicals (Toronto, Canada) was used. Plasma samples (150 μL) were mixed with 150 μL of 2 M trichloroacetic acid (TCA) and certifugated at 10,000 × g for 10 min. An aliquot of the supernatant was injected onto an analytical C_18_ reversed-phase column (Hypersil GOLD, 250 mm × 4.6 mm × 5 μm, Germany) maintained at 30 °C. The mobile phase constituted 0.6 % (v/v) orthophosphoric (V) acid and acetonitrile (50:50) at a flow rate of 1.0 ml/min. The elution profiles of propofol were monitored fluorometrically at an excitation wavelength of 270 nm and an emission wavelength of 310 nm. Plasma concentrations of propofol were determined by Chromeleon software version 6.80 (Dionex, Germany). For each analysis, the RSD (percentage of relative standard deviation) was calculated and for the HPLC/UV and fluorescence method, it was below 2.5 %. All samples were analysed in duplicate.

As the pharmacokinetic parameter, the mean retention time (MRT) was calculated for each patient using PKSolver software (Zhang et al. 2010). Observed MRT values were assigned to a percentile rank for a score of 25 and 75.

### Statistical analysis

All correlation analyses were performed using Student’s *t*-test for Pearson’s linear correlation coefficient, whereas correlation between metabolic profiles and genetic variants was proved using the Chi-squared and Fisher’s tests. The value indicating statistical significance was set at *p* ≤ 0.05. All calculations were performed using STATISTICA 12.0 software (StatSoft).

## Results

A total of 85 individuals were successfully screened for genetic variants p.Q172H (c.516G>T) in the *CYP2B6*, p.M33T (c.98T>C) in the *UGT1A9* and p.I359L (c.1075A>C) in the *CYP2C9* genes, using pyrosequencing.

The results showed that allele CYP2B6*9 (c.516T) was present in the study group with a frequency of 18 %, while the frequencies of alleles UGT1A9*3 (c.98C) and CYP2C9*3 (c.1075C) were 2% and 4.7%, respectively.

**Fig. 1:**
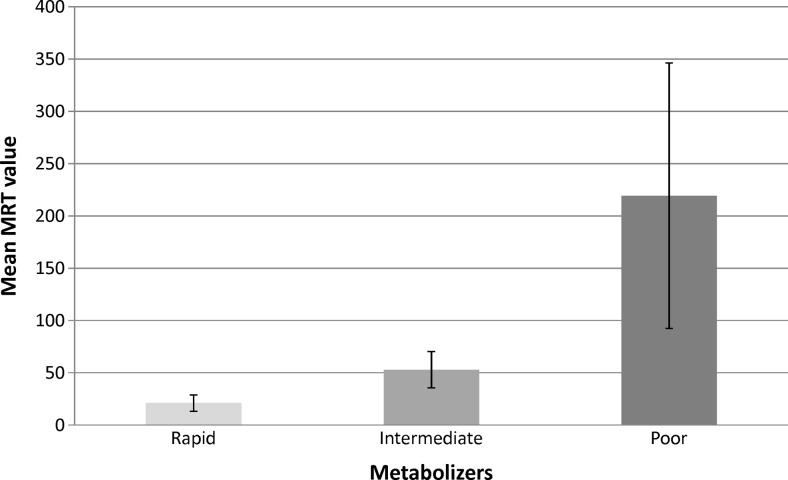
Characteristics of the propofol metabolisers group with mean retention time and standard deviation

Based on the plasma propofol concentration in five time points within 30 min after stopping anaesthetic infusion and on clinical data, the MRT was calculated for each patient (Table 3). We decided to use this independent pharmacokinetic parameter due to the proved high correlation and lack of statistically significant difference between MRT and t_1/2_. In our studied patients group, the MRT of propofol was in the range of 8–504 min. Using percentile rank, we identified poor (20), intermediate (42) and rapid (23) metabolisers of propofol, which constituted 24 %, 49 % and 27 % of the group, respectively (Fig. 1).

**Table 3:** Summary of the pharmacokinetic and genetic data

Patient number	Sex	Age	BMI	Total dose of propofol (mg)	Infusion time (min)	MRT (min)	*CYP2C9* c.1075A>C	*CYP2B6* c.516G>T	*UGT1A9* c.98T>C
1	M	46	29.8	300	65	49.8	AA	GT	TT
2	F	53	24.1	200	35	21.7	AA	GG	TT
3	M	52	30.9	500	10	178.7	AA	GG	TT
4	M	50	24.5	500	89	65.2	AC	GG	TT
5	M	37	24.7	700	59	35.8	AA	GG	TT
6	F	39	20.1	240	23	44	AA	GT	TT
7	F	56	37.2	130	14	10.5	AA	GG	TT
8	F	30	29.8	500	35	27.6	AA	GG	TT
9	M	30	25.8	600	33	37.9	AA	GG	TT
10	M	52	27.8	450	36	158.4	AA	GG	TT
11	M	31	23.6	360	22	47.5	AA	GG	TT
12	M	47	31.6	400	25	34	AA	GG	TT
13	M	51	44.8	430	23	37.3	AA	GG	TT
14	M	52	26.9	290	14	37.2	AA	GT	TT
15	F	53	31.3	570	45	98.4	AA	GG	TT
16	M	37	27.8	300	15	70.8	AA	GT	TT
17	M	51	26.3	550	49	48.3	AA	GG	TT
18	M	53	31.2	560	65	40.3	AC	GT	TT
19	F	49	37.0	400	24	467	AA	GG	TT
20	M	49	27.8	650	43	55.3	AA	GT	CT
21	F	48	23.5	350	18	25.9	AA	GG	TT
22	M	48	22.2	540	55	74	AA	GG	TT
23	M	52	20.9	300	20	33.8	AA	GG	TT
24	M	31	26.6	340	18	41.9	AA	GG	TT
25	F	32	21.3	340	22	54.7	AA	GT	TT
26	F	53	24.7	370	16	62.1	AA	GG	TT
27	F	52	23.1	330	13	59.5	AA	GG	TT
28	M	53	29.1	260	40	48.2	AA	GG	TT
29	M	49	30.7	500	25	28	AA	GT	TT
30	M	48	26.8	350	35	58	AA	GT	TT
31	M	52	22.9	430	15	15.8	AA	GT	TT
32	F	31	19.2	810	87	151	AA	GG	TT
33	M	51	30.0	780	45	29.2	AA	GG	TT
34	F	53	22.7	410	30	16.8	AA	GG	TT
35	M	35	35.2	840	53	198.9	AA	GG	TT
36	M	51	29.4	430	15	40.9	AA	GT	TT
37	F	46	24.8	650	87	43.8	AA	GG	TT
38	F	34	32.5	790	63	44.5	AA	GG	TT
39	M	48	26.2	310	33	19.4	AA	GT	TT
40	M	42	25.8	1150	67	106.5	AA	GG	TT
41	F	56	30.9	1230	108	92.9	AA	GG	TT
42	M	45	27.2	450	13	60.2	AA	GG	TT
43	M	44	26.3	360	10	82.5	AA	GT	TT
44	M	35	23.1	420	14	74.9	AA	GG	CT
45	M	41	29.6	700	18	108.5	AA	GT	CT
46	F	49	28.7	850	66	359	AA	GG	TT
47	F	53	29.4	600	25	149.7	AA	GT	TT
48	F	52	20.4	280	13	27.5	AA	GG	TT
49	M	38	27.8	570	15	8.4	AC	GG	TT
50	M	52	25.5	1160	89	128.7	AA	GG	TT
51	F	32	32.5	1340	142	208.3	AA	GG	TT
52	M	31	29.4	1150	63	379.9	AA	GG	TT
53	M	49	30.8	1300	63	100.7	AC	GT	CT
54	M	53	27.8	1892	132	202.6	AA	GG	TT
55	F	46	20.8	930	80	191.7	AA	GT	TT
56	F	45	29.4	1770	145	113.3	AA	GG	TT
57	F	36	25.9	1000	102	95.2	AA	GG	TT
58	M	50	39.5	1500	106	58.6	AA	GT	TT
59	M	31	26.5	550	45	29.2	AA	GT	TT
60	M	52	22.5	470	13	25	AA	TT	TT
61	M	52	27.5	900	24	32.4	AA	GG	TT
62	F	50	24.3	1840	136	70.8	AC	GG	TT
63	M	47	23.0	2200	113	169.9	AA	GG	TT
64	F	32	22.1	1350	118	61.2	AA	GT	TT
65	M	45	21.3	480	35	19.7	AC	TT	TT
66	F	46	37.0	200	18	23.3	AC	GG	TT
67	F	32	23.2	440	40	43.2	AA	GG	TT
68	M	38	29.0	1050	55	28.2	AC	GT	TT
69	M	52	29.8	420	11	29.1	AA	GT	TT
70	F	33	21.2	700	45	25.4	AA	GG	TT
71	M	31	28.8	900	45	393.6	AA	GG	TT
72	M	55	20.7	300	15	28.8	AA	TT	TT
73	M	43	26.8	1050	80	41.8	AA	GG	TT
74	M	32	22.4	1340	60	38.7	AA	GG	TT
75	F	33	23.0	270	15	28.5	AA	GG	TT
76	F	35	22.0	1600	95	58.9	AA	GT	TT
77	M	34	20.1	810	63	40.6	AA	GT	TT
78	M	32	25.2	830	65	54.9	AA	GG	TT
79	M	49	26.6	1200	67	116.6	AA	GG	TT
80	F	49	28.3	400	17	14.2	AA	GG	TT
81	F	38	27.7	500	13	37.8	AA	GT	TT
82	M	45	21.1	250	10	8	AA	GG	TT
83	M	43	29.4	320	13	8.2	AA	GG	TT
84	F	46	28.9	240	13	30.6	AA	GG	TT
85	M	54	34.7	1750	89	504.1	AA	GG	TT

On the basis of the Chi-squared and Fischer’s tests, we observed that homozygotes c.516T/T were statistically more often present in the rapid metabolisers group (*p* < 0.05) (Table 4). Furthermore, propofol MRT was correlated with the patient’s BMI (*p* < 0.05). The MRT was significantly longer in the case of individuals with a higher BMI. Moreover, we have observed that infusion time determines the MRT (*p* < 0.05). However, we did not report a correlation between Cmax and the MRT (*p* > 0.05). We also did not find the patient’s age to affect the pharmacokinetic marker MRT (*p* > 0.05). The infusion time did not influence the Cmax value (*p* > 0.05).

**Table 4:** Comparison of genotypes distribution among the patients group with different pharmacokinetic profiles

Sequence change	Genotype	Poor metabolisers		Intermediate metabolisers		Rapid metabolisers		*p*-Value
		*n*	%	*n*	%	*n*	%	
*CYP2C9* c.1075A>C (p.Ile359Leu)	AA	18	90	39	93	20	87	0.99
	AC	2	10	3	7	3	13	
	CC	0	0	0	0	0	0	
*CYP2B6* c.516G>T (p.Gln172His)	GG	16	80	27	64	14	61	0.03*
	GT	4	20	15	36	6	26	
	TT	0	0	0	0	3	13	
*UGT1A9* c.98T>C (p.Met33Thr)	TT	18	90	40	95	23	100	0.35
	TC	2	10	2	5	0	0	
	CC	0	0	0	0	0	0	

*Statistically significant

## Discussion

Understanding the factors, especially genetic polymorphism, that influence the required personalised dose of propofol in general anaesthesia was the goal of the present study. Justification for our investigation was provided by ambiguous literature data concerning the participation of *CYP2B6* and *UGT1A9* polymorphisms in propofol metabolism. We have analysed the plasma pharmacokinetic profile of propofol in 85 patients after a stopped infusion of anaesthetic with an average dose of 2.5 mg/kg. As a parameter describing the pharmacokinetics in each patient, the MRT was finally calculated. A high inter-individual variability of the MRT has allowed for the identification of poor, intermediate and rapid metabolisers (Fig. 1).

Analysis of the genotype distribution (for positions c.516 in the *CYP2B6*, c.98 in the *UGT1A9* and c.1075 in the *CYP2C9* genes) in all pharmacokinetic profiles showed that only the change c.516G>T correlates with the propofol biotransformation rate. Homozygotes c.516T/T were statistically more often identified in rapid-metabolising individuals.

Our results confirm the significance of this non-synonymous substitution c.516G>T of the *CYP2B6* gene in the propofol metabolic rate and further dosing, which was proved in several previous studies (Kansaku et al. 2011; Mastrogianni et al. 2014; Mourão et al. 2016). Kansaku et al. (2011) has proved this change as a genetic factor determining the pharmacokinetics and pharmacodynamics of propofol. They correlated a high maximum blood concentration (Cmax) of anaesthetic with genotype c.516T/T. It may suggest, in contrast to our study, a poor metabolism of propofol. This sequence variation c.516G>T was also the subject of pharmacokinetic research on a group of Greek women. Allele c.516T determined a high blood level of propofol, and its frequency was 29.5% (Mastrogianni et al. 2014). A recent study conducted by Mourão et al. (2016) shared the conclusions formulated by Kansaku et al. (2011) and Mastrogianni et al. (2014), suggesting that allele c.516T determines a lower dose of propofol administered to patients undergoing intravenous general anaesthesia.

On the other hand, Iohom et al. (2007) was the first to suggest an important role of the *CYP2B6* gene in the individual pharmacokinetic and pharmacodynamic profiles of propofol. However, they did not demonstrate a correlation between change p.Q172H and clearance of propofol. Similar conclusions were reached in studies performed by Khan et al. (2014); none of the analysed polymorphisms in *CYP2B6* were associated with a propofol response. Also, Loryan et al. (2012) did not prove a significant linkage between *CYP2B6* and *UGT1A9* allelic variants and blood propofol concentration. As they explained, for some of the rare genetic polymorphisms, the study group size was probably too small.

Among the clinical parameters collected in our study, only BMI was significantly correlated with the pharmacokinetic profiles of propofol. A longer retention time observed in patients with higher BMI explains the lipophilic nature of the anaesthetic (Lotia and Bellamy 2008). However, we did not confirm the conclusion propounded by Loryan et al. (2012) concerning the impact of sex on propofol metabolism.

The analysed allele CYP2C9*3 (p.I359L), although it is known as being associated with altered enzyme activity, did not have a significant effect on the biotransformation rate of propofol in our study group. We demonstrated this allele frequency of 4.7 %, which corresponds to the range reported in Caucasians. Global studies proved the allele CYP2C9*3 to be correlated with the overdose risk of warfarin and phenytoin (Lindh et al. 2009). Because, so far, there are no data regarding the role of p.I359L change in the *CYP2C9* gene in propofol metabolism in anaesthetised patients, it is difficult to discuss the outcome. Certainly, an important explanation for our results may constitute suggested substrate dependence of the *CYP2C9* polymorphism.

The effect of the *CYP2B6* p.Q172H change on the propofol pharmacokinetic profile reported in the available studies is not fully elucidated. Nevertheless, CYP2B6 plays an important role in the biotransformation process of this anaesthetic by the hydroxylation pathway. Possibly, in our study group, glucuronidation may be the main reaction in anaesthetic metabolism, which would minimise a significant influence of *CYP2B6* gene polymorphism in the propofol response. On the other hand, there are certain differences between parameters in our study and opposed research performed by Kansaku et al. (2011). The average age of patients, as well as the infusion time of propofol, was higher in the Japanese investigation (65 years; an average of 250 min), which may partly explain the significant divergences in the obtained results. Moreover, the analysis time of propofol clearance in our research was limited to the first 30 min after the end of propofol infusion, while in the Japanese study, it reached 60 min. A clearer demonstration of the influence of the *CYP2B6* c.516G>T mutation on propofol concentration in patient plasma would probably be possible with the use of the determination of propofol’s metabolites; for example, propofol glucuronide and 4-hydroxypropofol. Additionally, the low frequency of the c.516G>T variant of the *CYP2B6* gene may be a source of discrepancies between the studies. Kansaku et al. (2011) found two patients as c.516T homozygotes (of the group of 61 patients) and classified them as poor metabolisers, whereas in our study, three patients were identified as homozygotes TT; however, they were all classified as rapid metabolisers. The statistical analysis has shown the significant correlation of this genotype with a high rate of propofol metabolism.

We can conclude that polymorphism c.516G>T in the *CYP2B6* gene and BMI affect the metabolism rate of propofol. Our results constitute an inspiration for further extensive studies including metabolites measurements and larger groups of patients. It is suggested that there are more candidate genes as genetic determinants of individual propofol response, such as genes coding for transporters and receptor proteins (Iohom et al. 2007). By using a valuable tool of molecular biology, high-throughput sequencing techniques, which enable efficient and deep multi-gene analysis, it seems possible to be able to deliver to clinicians the outline for optimal anaesthesia with propofol to avoid the risk of adverse reactions (Pareek et al. 2011).
